# Editorial: Women in Science—Hematology 2021

**DOI:** 10.3389/fmed.2022.926204

**Published:** 2022-06-13

**Authors:** Eleni Gavriilaki, Chien-Ling Huang, Lalitha Nayak

**Affiliations:** ^1^Hematology Department—Bone Marrow Transplantation Unit, G Papanicolaou Hospital, Thessaloniki, Greece; ^2^Department of Health Technology and Informatics, The Hong Kong Polytechnic University, Hong Kong, Hong Kong SAR, China; ^3^Division of Hematology and Oncology, University Hospitals, Case Western Reserve University, Cleveland, OH, United States

**Keywords:** medicine, hematology, STEM, histo-hematology, molecular-hematology, UNESCO

Although the proportion of women and men in science, technology, engineering, and mathematics (STEM) at undergraduate level is relatively equal, there is a lack of representation of women in senior positions in public health. According to the UNESCO Institute for Statistics (UIS) data in 2016, <30% of researchers in STEM are women. In the field of hematology, there are many highly influential and successful women who are contributing to the field and tackling important questions. Yet, female scientists are still under-represented in various aspects of academic life. More importantly, the COVID-19 (coronavirus disease 2019) pandemic had a significant impact on female scientists and especially early career researchers in hematology (1, 2). In this context, maternity issues remain an important aspect, especially pregnancy and lactation. Since the latter by no means can be shared with husband/spouse, some female colleagues consider them as a privilege, whereas others as an issue that significantly affects their career.

Several initiatives have been recently created to increase the visibility of women in science, such as awards for women in STEM and diversity initiatives like those of the European Hematology Association (EHA). However, evidence indicates that a gender bias is still present throughout many scientific disciplines.

This Research Topic highlighted female contributions to medicine, specifically in the field of hematology, aiming to delineate:

- General perspectives on a specific field of research inspired, started, or sparked by a woman
- Articles celebrating outstanding female researchers and their contributions to computer science and public health
- Public health studies led by women researching technology and health.

To be considered for this Research Topic, the first or last authors were female researchers, and we recommend early career researchers to team up with senior female colleagues. All articles submitted to us for this Research Topic underwent a rigorous peer review process. Ultimately, 11 articles were published.

(i) The Spanish group led by Ceballos et al. published a brief research report on coagulation markers in COVID-19 suggesting that lower levels at diagnosis might be associated with higher morbidity and mortality.
(ii) In the context of COVID-19, Anipindi et al. documented an interesting case report of cerebral venous sinus thrombosis.
(iii) In a collaboration of first and last female authors, Vanegas et al. provided a mini review article on unrelated umbilical cord blood graft vs. haploidentical donor transplantation.
(iv) In the life-threatening field of thrombotic microangiopathies, Pang et al. identified a rare case of thrombotic microangiopathy induced by remethylation disorders.
(v) In their very interesting paper, Fenoglio et al. from Italy, report on a prospective single study evaluating the clinical presentation and effects of rituximab in patients with non-HCV-related cryoglobulinemic syndrome.
(vi) This study from Palestine, led by Aldwaik et al. describes the characteristics and evaluates hematological, biochemical, and hormonal findings in β-thalassemia patients in the West Bank and highlights the importance of establishing patient-tailored comprehensive assessment and follow-up protocols with emphasis on blood transfusion and iron-chelation practices for the management of this disease.
(vii) Xu et al. demonstrate how the addition of two condition-specific bolt-on items can increase performance on the EQ-5D-5L in patients with hemophilia.
(viii) Patterson et al. sought to assess thalassemia patients' knowledge of transfusion, complications, and guidelines. Their results suggest that the necessity of increased patient education on terminologies pertaining to red cell transfusion and implementation of a nationwide registry to make transfusion data available to providers are some of the actions that could help reduce transfusion complications,
(ix) Zhang et al. show that the presence of circulating nucleated red blood cells (NRBCs) in patients of hemorrhagic fever with renal syndrome is associated with disease severity. This study provides further insights on the role of and pathological changes in NRBCs during Hantaan virus infection.
(x) An interesting case report, published by Sun et al., reported on gestational psittacosis concomitant with secondary hemophagocytic syndrome. Metagenomic next-generation sequencing is recommended to provide a clear diagnosis in addition to hematological examinations.
(xi) In a novel methods article, a modified preparation method of an ideal platelet-rich fibrin matrix from whole blood has been described (Reksodiputro et al.).

Taking into account the multi-disciplinary character of this Research Topic, we hope that it will inspire female researchers and clinicians to continue their explorations into novel advances in their fields.

## Author Contributions

All authors listed have made a substantial, direct, and intellectual contribution to the work and approved it for publication.

## Funding

EG was supported by the ASH Global Research Award. C-LH was supported by the Health and Medical Research Fund Commissioned Research on COVID-19 (COVID1903007). LN was supported by a National Heart, Lung, and Blood Institute grant HL121131-01.

## Conflict of Interest

EG has received honoraria from Alexion, Gilead, Sanofi, Sobi, and Omeros Pharmaceuticals. The remaining authors declare that the research was conducted in the absence of any commercial or financial relationships that could be construed as a potential conflict of interest.

## Publisher's Note

All claims expressed in this article are solely those of the authors and do not necessarily represent those of their affiliated organizations, or those of the publisher, the editors and the reviewers. Any product that may be evaluated in this article, or claim that may be made by its manufacturer, is not guaranteed or endorsed by the publisher.
